# Association of Genetic Markers with CSF Oligoclonal Bands in Multiple Sclerosis Patients

**DOI:** 10.1371/journal.pone.0064408

**Published:** 2013-06-13

**Authors:** Maurizio A. Leone, Nadia Barizzone, Federica Esposito, Ausiliatrice Lucenti, Hanne F. Harbo, An Goris, Ingrid Kockum, Annette Bang Oturai, Elisabeth Gulowsen Celius, Inger L. Mero, Bénédicte Dubois, Tomas Olsson, Helle Bach Søndergaard, Daniele Cusi, Sara Lupoli, Bettina Kulle Andreassen, the International Multiple Sclerosis Genetics Consortium, the Wellcome Trust Case Control Consortium 2, Kjell-Morten Myhr, Franca R. Guerini, the PROGEMUS Group, the PROGRESSO Group, Giancarlo Comi, Filippo Martinelli-Boneschi, Sandra D'Alfonso

**Affiliations:** 1 MS Centre, SCDU Neurology, AOU Maggiore della Carità, Novara, Italy; 2 Interdisciplinary Research Center of Autoimmune Diseases (IRCAD), University of Eastern Piedmont, Novara, Italy; 3 Department of Health Sciences, University of Eastern Piedmont, Novara, Italy; 4 Department of Neurology & Laboratory of Neurogenetics of complex disease, CNS Inflammatory Unit, Institute of Experimental Neurology, Scientific Institute San Raffaele, Milan, Italy; 5 Department of Neurology, Oslo University Hospital, Ullevål, Oslo, Norway; 6 Institute of Clinical Medicine, University of Oslo, Oslo, Norway; 7 Laboratory for Neuroimmunology, Section of Experimental Neurology, KU Leuven, Leuven, Belgium; 8 Department of Clinical Neurosciences, Centre for Molecular Medicine CMM, Karolinska Institutet, Karolinska Hospital, Stockholm, Sweden; 9 Danish Multiple Sclerosis Center, Copenhagen University Hospital, Rigshospitalet Copenhagen, Denmark; 10 Department of Health Sciences, University of Milano, Milan, Italy; 11 Department of Clinical Molecular Biology and Laboratory Sciences (EpiGen) and Department of Biostatistics, University of Oslo, Oslo, Norway; 12 The Norwegian Multiple Sclerosis Registry and Biobank, Department of Neurology, Haukeland University Hospital, Bergen, Norway; 13 The KG Jebsen Centre for MS-research, Department of Clinical Medicine, University of Bergen, Bergen, Norway; 14 Fondazione Don C.Gnocchi, IRCCS S. Maria Nascente, Milan, Italy; Institute Biomedical Research August Pi Sunyer (IDIBAPS) – Hospital Clinic of Barcelona, Spain

## Abstract

**Objective:**

to explore the association between genetic markers and Oligoclonal Bands (OCB) in the Cerebro Spinal Fluid (CSF) of Italian Multiple Sclerosis patients.

**Methods:**

We genotyped 1115 Italian patients for HLA-DRB1*15 and HLA-A*02. In a subset of 925 patients we tested association with 52 non-HLA SNPs associated with MS susceptibility and we calculated a weighted Genetic Risk Score. Finally, we performed a Genome Wide Association Study (GWAS) with OCB status on a subset of 562 patients. The best associated SNPs of the Italian GWAS were replicated *in silico* in Scandinavian and Belgian populations, and meta-analyzed.

**Results:**

HLA-DRB1*15 is associated with OCB+: p = 0.03, Odds Ratio (OR) = 1.6, 95% Confidence Limits (CL) = 1.1–2.4. None of the 52 non-HLA MS susceptibility loci was associated with OCB, except one SNP (rs2546890) near *IL12B* gene (OR: 1.45; 1.09–1.92). The weighted Genetic Risk Score mean was significantly (p = 0.0008) higher in OCB+ (7.668) than in OCB− (7.412) patients. After meta-analysis on the three datasets (Italian, Scandinavian and Belgian) for the best associated signals resulted from the Italian GWAS, the strongest signal was a SNP (rs9320598) on chromosome 6q (p = 9.4×10^−7^) outside the HLA region (65 Mb).

**Discussion:**

genetic factors predispose to the development of OCB.

## Introduction

The presence of oligoclonal bands (OCB) in the Cerebro Spinal Fluid (CSF) is a distinctive hallmark of Multiple Sclerosis (MS), found in 48 to 100% of patients in European populations [1]. The presence of a genetic influence on the OCB phenotype is suggested by its association, in several populations, with HLA-DRB1*15, which is also the strongest genetic susceptibility factor for the development of the disease. The Odds Ratio (OR) for OCB positivity ranges from 1.6 [2] to 3.4 [3], not dissimilarly from the OR observed for disease susceptibility. In spite of this indication and of all the efforts undertaken in the study of MS genetic susceptibility, the genetic association with the OCB phenotype was not extensively investigated so far. We aimed to explore the association between OCB and other genetic markers in Italian MS patients, both with a classical unbiased, genome-wide strategy and with a special attention for known MS susceptibility markers. This approach was worth attempting given the preliminary data on HLA-DRB1*15. We utilized an Italian population as a discovery sample for genome-wide study, and replicated the new findings in independent European cohorts from Scandinavia and Belgium.

## Materials and Methods

### Ethics Statement

The PROGEMUS study was approved by the Ethical Committee of the AOU Maggiore della Carità, Novara (Coordinating Centre) and those of the following Institutions: IRCCS Fondazione Cà Granda, Ospedale Maggiore Policlinico, Milan, Fondazione Istituto Neurologico C. Mondino-IRCCS, Pavia, ASO S. Luigi Gonzaga, Orbassano, Fondazione Don C.Gnocchi, IRCCS S. Maria Nascente, Milan, AO S. Croce e Carle, Cuneo, AUSL Valle d'Aosta, Aosta, AOU S.Giovanni Battista, Turin, ASL-TO2, Turin, AOU Integrata, Verona, AO S. Andrea, Rome, Italy. The PROGRESSO study was approved by the Ethical Committee of the San Raffaele Hospital, Milano (Coordinating Centre) and those of the following Institutions: AO Spedali Civili, Brescia, AO S. Antonio Abate, Gallarate, San Carlo Hospital, Potenza, Italy. The Danish study was approved by the Danish Ethical Committee Review Board for Copenhagen and Frederiksberg/today The Capital Region of Denmark. The Norwegian study was approved by The Regional Committee for Medical and Health Research Ethics (REC)- South East and REC West, Norway. The Swedish study was approved by the Regional Ethical Review Board in Stockholm. The Belgian study was approved by the Commissie Medische, Ethiek/Toetsingscommissie University Hospital, Leuven. All individuals involved in this study signed a written informed consent.

### Patients

A total of 1347 consecutive Italian MS patients diagnosed according to McDonald criteria [4], were ascertained by two Italian consortia, PROGEMUS (N = 1002), and PROGRESSO (N = 345). Patients born in Sardinia, of non-Italian ancestry, or refusing permission to DNA testing were not ascertained. A lumbar puncture was performed in 1115 patients (83% of the original cohort), 731 women and 384 men. Excluded patients (N = 232) were not statistically different from those included with regards to gender, clinical form (bout onset vs. primary progressive), age at onset, and MSSS [5] (data not shown). OCB positivity (OCB+) was defined as two or more bands present in CSF but absent in plasma at the same point in time. When two CSF analyses were performed, we considered the result of the last one. In Italy, OCB were assessed in laboratories pertaining to the network of Italian certified Neuroimmunology Laboratories and analyzed by isoelectric focusing and immunodetection of IgG. In Belgium, Norway and Sweden (apart from Stockholm hospitals) CSF analysis was performed with agar electrophoresis before 2000–2 and with isoelectric focusing thereafter. In the Stockholm area, CSF analysis was done according to Olsson et al. [6]. All the samples and the clinical information were collected after written informed consent. The clinical characteristics of the Italian, Belgian, and Scandinavian MS patient samples are described in Table 1.

**Table 1:** Clinical characteristics of MS patients according to OCB status in the three populations.

Characteristic	OCB negative			OCB positive		
	Italy	Belgium	Scandinavia	Italy	Belgium	Scandinavia
N (%)	150 (13%)	39 (11%)	168 (11%)	965 (87%)	317 (89%)	1406 (89%)
Male/female ratio	0.81	0.95	0.51	0.76	0.47	0.39
% PP – MS	15%	18%	10%	19%	12%	9%
Mean age at onset	33.8	36.1	34.7	31.7	33.3	33.0
Mean [14]	2.7	4.6	2.9	3.3	4.4	2.9
Mean MSSS [15]	3.9	5.5	4.0	4.2	5.4	3.9

*Abbreviations:* **PP**  =  Primary Progressive, **OCB**  =  oligoclonal bands, **EDSS**  =  Expanded disability status scale, **MSSS**  =  Multiple sclerosis severity score.

### Genetic analysis

**a) overview**

HLA-DRB1*15 and HLA-A*02 genotyping was performed in 1115 Italian patients, as previously described [7]. DRB1*03 genotypes were imputed by HLA*IMP software [8]. We tested 925 patients (814 OCB+, 111 OCB−) for association with the 52 non-HLA SNPs, shown to be associated with MS susceptibility in the recent large IMSGC-WTCCC2 genome wide association study (GWAS) [9]. In the same Italian group we calculated a weighted Genetic Risk Score (wGRS), as described by De Jager [10], using the ORs [9] for the 52 non-HLA MS susceptibility variants, and three HLA alleles (DRB1*15, DRB1*03 and A*02). Finally, we performed a GWAS with OCB status on the subset of Italian patients from the IMSGC-WTCCC GWAS study [9]. This sample included 562 (513 OCB+ and 49 OCB−) patients, genotyped with the Illumina 660-Quad platform. After quality control (QC) filtering [9], there were 504967 SNPs remaining. The genomic inflation factor was 1.009. The same (QC) filtering [9] has been applied to the Scandinavian and Belgium datasets. The association of each of the above markers with OCB status was evaluated comparing the allele frequency in OCB+ vs. OCB− patients. All GWAS statistical analyses (QC, association and meta-analysis) were performed with PLINK software.

**b) Statistical and computational details**

*wGRS calculation:* The wGRS was formulated according to De Jager et al. [10] using the following model: wGRS = Σ (n_risk alleles_ * lnOR). We used a total of 55 genetic risk loci (3 HLA and 52 non-HLA loci). The 52 non-HLA loci included 23 well known MS loci, previously identified in several large-scale association studies and validated in the recent IMSGC and WTCCC GWAS [table S2 in reference 9], and 29 new loci, whose association with MS have been reported for the first time in the same GWAS [table S3 in reference 9]. The 3 HLA markers were classical HLA alleles (namely HLA-DRB1*15:01, HLA-DRB1*03:01 and HLA-A*02:01) showing a well established association with MS. For each marker, the OR used in the model is the allelic OR from IMSGC and WTCCC GWAS, discovery plus validation set [9].

**c) GWAS analysis**

Quality Controls (QC) on the GWAS data for all datasets were performed utilizing the filters reported in reference 9. Briefly, for individual QC: call rates >92.5%, relatedness <5% IBD (Identity by descent), for SNP QC: MAF>0.10, call rate >0.90, Hardy-Weinberg >10^−50^.

**d) GWAS analysis: Association and Meta-Analysis**

Basic association testing was performed with the command line: plink – assoc – ci 0.95– reference-allele <file.txt>. To select the covariates to be used in a logistic analysis, we checked the following potential confounders or interactors in the discovery dataset from the Italian population: gender, age of onset and MS clinical subtype (Primary Progressive vs. Bout Onset) for association with OCB status, using basic Chi-square test (for gender and clinical subtype) or Student's T test (for age of onset). None of these factors was differentially distributed in OCB+ vs. OCB− patients, therefore none of these variables were selected as covariates.

Population stratification was evaluated both with the Genomic Control calculation (λ), and by means of the quantile-quantile distribution. The λwas evaluated with PLINK, by adding – adjust to the basic association command line. The quantile-quantile distribution was visually analysed by Q–Q plot, generated by the R software ( Figure S1) The genomic inflation factor (λ) was very low (1.009), indicative of a reduced population stratification. This is confirmed also by the quantile-quantile distribution (Figure S1). On these bases, and on the bases of the previous analysis on covariates, we decided not to add Principal Components in our model, and therefore not to implement a logistic analysis. The Manhattan plot was created with the software Haploview, providing the.assoc file.

Genetic and clinical data for Scandinavian and Belgian patients were analyzed with a similar pipeline.

Meta-analysis was performed with PLINK software, basic option (string: plink – meta-analysis), that uses, for all datasets, the following information: SNP ID, chromosome, position, alleles, OR, standard error of OR and p-value. We calculated ORs and p-values using both fixed and random effects models (reported in Table S1). Forest plot was instead created with R software, library “meta”.

## Results

**Figure 1:**
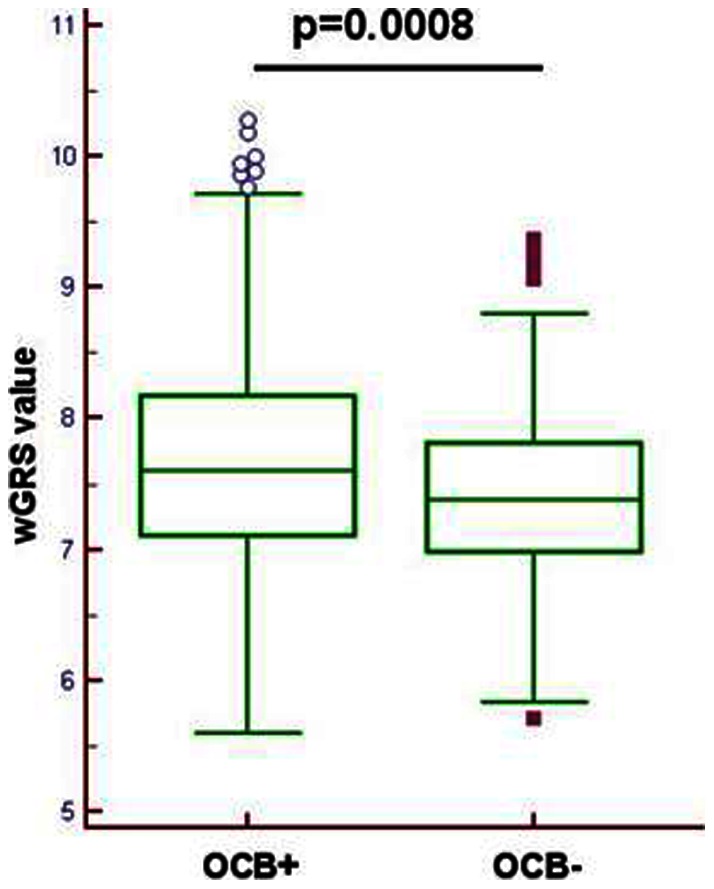
Distribution of the wGRS in MS patients positive (OCB+) or negative (OCB−) for OCB. wGRS (weighted Genetic Risk Score) has been calculated using the ORs for the 52 non-HLA MS susceptibility variants,^6^ and three HLA alleles (DRB1*15, DRB1*03 and A*02). The reported p value derived from the comparison of the mean wGRS (Student's t test) in OCB+ vs OCB−.

**Figure 2:**
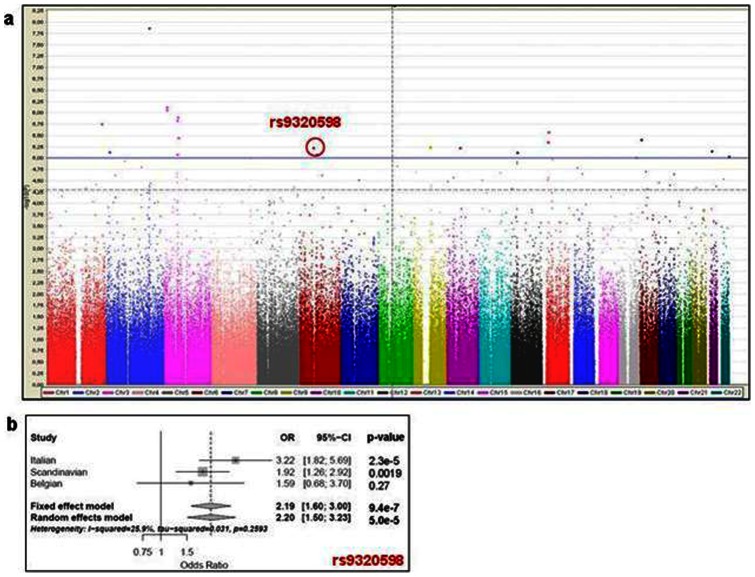
Results of the GWAS for OCB status and meta-analysis after *in silico* replication. (**a**) Manhattan Plot of the GWAS performed in the Italian OCB+ vs OCB− MS patients, as a discovery dataset. The blue horizontal line indicates the threshold of p values arbitrarily chosen to select the “best associated SNPs” (p-value <10^−4^, 89 SNPs) to perform *in silico* replication (Table S1) using data from Scandinavia and Belgium populations. (**b**) Meta-analysis for the rs9320598 SNP, the strongest signal after the meta-analysis of the combined results of all datasets (Italy, Scandinavia and Belgium). The forest plot summarizes the results obtained for the discovery Italian dataset, for the replication datasets from Scandinavia and Belgium, and the combined analysis calculated using the random and fixed-effect method.

Our results confirm that HLA DRB1*15 is positively associated with OCB+ (p = 0.03 OR = 1.6; 95%CL = 1.1–2.4); 313 patients were DRB1*15+/OCB+, 35 DRB1*15+/OCB−, 652 DRB1*15−/OCB+, and 115 DRB1*15−/OCB−. None of the 52 non-HLA MS susceptibility loci (Table S2) was associated with OCB status, except one SNP (rs2546890) near *IL12B* gene, with the MS risk allele conferring an OR of 1.45 (95% CL 1.09–1.92) for OCB positivity (p = 0.010). However, the combined effect of these markers plus three HLA MS risk alleles (DRB1*15, DRB1*03 and A*02) on OCB status (evaluated constructing a wGRS) was significant. In fact, the wGRS mean was significantly (p = 0.0008) higher in OCB+ (7.668) than in OCB− (7.412) patients (Figure 1) even after removing DRB1*15 (p = 0.0096). After removing the three HLA MS risk alleles, a similar trend, although non significant (p = 0.06), was still observed (wGRS mean: 6.784 in OCB+ vs. 6.686 in OCB−). Finally, to search for new associations, not yet correlated with disease susceptibility, we performed a GWAS with OCB status using the PROGEMUS/PROGRESSO subset of the IMSGC-WTCCC GWAS [9] as a discovery sample (Figure 2) to perform a GWAS with OCB status. Replication of the best signals in the Italian dataset was performed in *silico* using data from the previously published GWAS [9] from independent populations from Scandinavia (Norwegian-Danish-Swedish) (1367 OCB+ and 161 OCB−) and Belgium (317 OCB+ and 39 OCB−). To select SNPs for replication, an arbitrary significance threshold (p-value <10^−4^) was chosen as a balance between sensitivity and specificity in order to ensure the detection of genuine associations that may not reach GWAS threshold in our sample set. A total of 89 SNPs were selected (Table S1). After the meta-analysis of the combined datasets (Italy, Scandinavia and Belgium), the strongest signal was a non-HLA SNP (rs9320598) on chromosome 6 (p = 9.4×10^−7^) (Figure 2, Table S1) outside the HLA region (65 Mb).

## Discussion

The percentage of OCB− patients (12%) in our Italian sample was in the range of that found in South Europe and OCB status was clearly associated with carriage of DRB1*15 as in other studies [1]. The intrathecal synthesis of immunoglobulins, as marked by OCB status, is observed in the preclinical phase of MS and the possibility of conversion from negative to positive is low [1], suggesting that there is a genetic trait predisposing to its development. Three findings from our data support this hypothesis: 1) DRB1*15 is associated with OCB+; 2) the combination of non-HLA markers associated with MS susceptibility also showed a trend for association with the presence of OCB as suggested by our wGRS, although most of its predictive power comes from DRB1*15; 3) after meta-analysis of the Italian best associated markers with two independent data-sets, we found a non-HLA SNP on chromosome 6 that almost reached GWAS significance. This SNP maps at 300 kb telomeric to C6orf167/MMS22L, a gene involved in the regulation of genome stability and DNA repair [11], and at 400 kb telomeric to KLHL32/KIAA1900, a gene with still uncharacterized function. Since this association did not reach genome-wide significance, further independent studies are needed to confirm this finding. Although it may be premature to assume that this locus is associated with OCB status, this and the other findings in our study suggest that genetic factors may be involved in the development of OCB. OCB status has been associated in turn with MS prognosis [12]; thus our findings could provide some clues to clarify the genetic predictors of disease prognosis.

One possible bias of our study is the selection of MS patients that underwent lumbar puncture; however their percentage was high and the clinical characteristics of those with CSF examination were similar to those without. We cannot exclude that the group of OCB negative patients represent a peculiar type of MS or even not MS cases. However, the clinical similarity of OCB+ and OCB− patients argues against this [13].

In conclusion, our data support the hypothesis that HLA genes and possibly other non-HLA genes predispose to the development of OCB.

## Supporting Information

Figure S1 **Quartile-Quartile (QQ) plot for the GWAS analysis in the Italian population.**
(TIF)

Table S1 (XLSX)

Table S2 (XLS)
