# Supplementary figures and images for: Association of Genetic Markers with CSF Oligoclonal Bands in Multiple Sclerosis Patients
Source: PLoS One. 2013 Jun 13;8(6):e64408. doi: 10.1371/journal.pone.0064408 (PMC3681825; doi:10.1371/journal.pone.0064408)

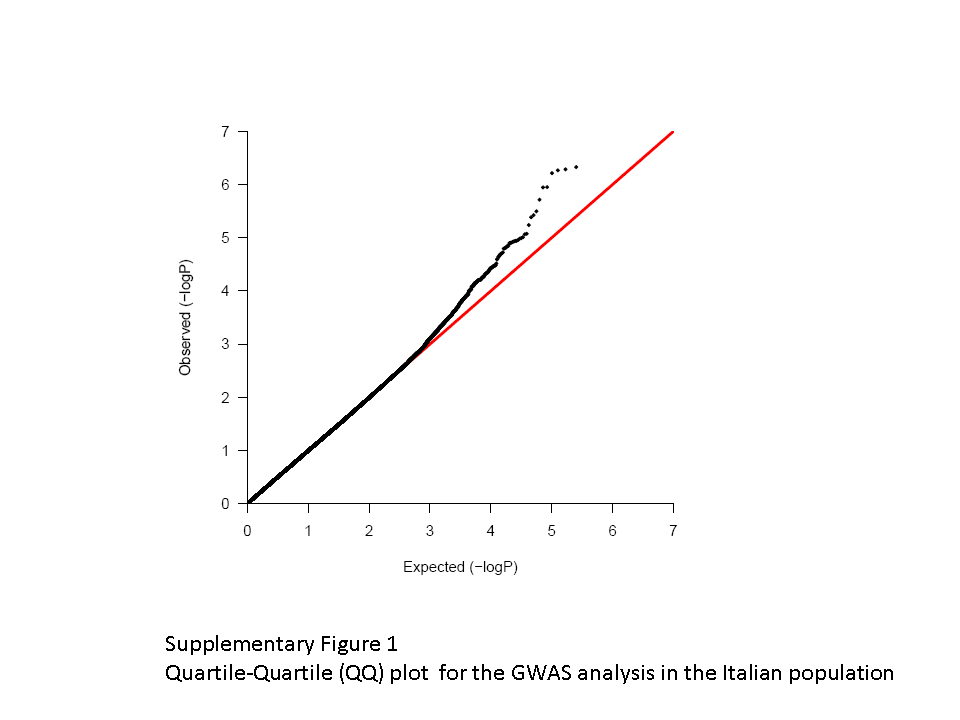

Supplement: Figure S1 — Quartile-Quartile (QQ) plot for the GWAS analysis in the Italian population. (TIF) [file pone.0064408.s001.tif]
